# No effect of an oleoylethanolamide-related phospholipid on satiety and energy intake: a randomised controlled trial of phosphatidylethanolamine

**DOI:** 10.1186/1476-511X-7-41

**Published:** 2008-10-29

**Authors:** FE Lithander, CM Strik, A-T McGill, AK MacGibbon, BH McArdle, SD Poppitt

**Affiliations:** 1 Human Nutrition Unit, University of Auckland, New Zealand; 2 School of Biological Sciences, University of Auckland, New Zealand; 3 School of Medical Sciences, University of Auckland, New Zealand; 4 School of Population Health, University of Auckland, New Zealand; 5 Department of Statistics, University of Auckland, New Zealand; 6 Fonterra Research Centre, Palmerston North, New Zealand; 7 Department of Clinical Medicine, Trinity College Dublin, Dublin, Ireland

## Abstract

**Background:**

Phosphatidylethanolamine (PE) is a phospholipid which is biosynthesized into long chain N-acylethanolamines (NAEs) including oleoylethanolamide (OEA), a known inhibitor of food intake. The aim of this study was to investigate whether PE-containing lipids can also inhibit intake. This was a 4 treatment intervention where 18 male participants were given a high-fat test breakfast (2.5MJ, 53 en% fat) containing (i) high-phospholipid, high-PE lipid (ii) high-phospholipid, medium-PE lipid (iii) no-phospholipid, no-PE control lipid or (iv) water control, in a randomised cross-over. Visual analogue scales (VAS) were used to assess post-ingestive hunger and satiety, and energy intake (EI) was measured at an ad libitum lunch meal after 3.5hours.

**Results:**

When compared with the water control, the 3 lipid treatments resulted in lower levels of hunger and thoughts of food, greater fullness and satisfaction (all, treatment*time interaction, P<0.001), and a lower EI (P<0.05). However, there was no difference in any of the VAS measures when the 2 PE lipid treatments were compared with no-PE control lipid, nor when medium-PE was compared with high-PE. Unexpectedly participants ate significantly more energy at the lunch meal when the 2 PE lipid treatments (medium-PE:5406 kJ, 334 sem; high-PE:5288 kJ, 244 sem) were compared with the no-PE control lipid (5072 kJ, 262 sem, P<0.05), although there was no dose effect between the medium- and high-PE treatments.

**Conclusion:**

Despite the close relationship of PE with OEA, there was no evidence from this acute study that dietary phospholipids containing PE can favourably modify eating behaviour.

## Introduction

The inability to control energy intake (EI), compounded by low levels of physical activity, appears key to the development of obesity. Regulation of appetite is central to the control of weight gain, and identifying foods which can modulate hunger and satiety and hence decrease intake is an area of considerable interest. There is a body of data which suggests that dietary lipids may be the least satiating of the 3 food macronutrients [1-4], but that the physicochemical properties such as fatty acid saturation [5-7], fatty acid chain length [8], and fat emulsification and intestinal delivery rates [9-12] may all affect the degree to which lipids can control hunger. Given the sensory appeal of dietary fat and the fact that it comprises such a high percentage of the Western diet (35–45% energy), the identification of highly satiating lipid products would be attractive.

Phospholipids (PL) are crucial lipid components of biological membranes and, whilst present in the diet in much smaller gram amounts than triglycerides, are found typically in items including eggs, dairy, liver and oils such as soybean. Whilst phosphatidylcholine (PC, also known as lecithin) is the most abundant PL in animal tissues [13], phosphatidylethanolamine (PE) is also found in biological membranes and hence is also consumed within our diet. PLs are commonly used as emulsifiers within the food industry. Of particular interest in the area of appetite regulation is PE which is known to be metabolized endogenously into a range of ethanolamides of long chain fatty acids, collectively termed the *N*-acylethanolamines (NAEs), and which include *N*-arachidonoylethanolamine (anandamide) and its analogue oleoylethanolamide (OEA) [14]. OEA is a lipid which has become prominent as an established inhibitor of food intake in animal models and which appears to regulate feeding through activation of the nuclear receptor peroxisome-proliferator-activated receptor-α (PPAR-α) [15,16]. OEA decreases food intake by as much as 40–70% when introduced both by intra-peritoneal injection [15,17] and orally [18] into long-term fasted animals. Hence OEA has become a possible therapeutic target for modification of food intake and subsequent weight loss in humans. Effects of OEA are rapid with significant changes in food intake observed 60 minutes post-dose [18] and maintained over a period of 12 hours.

In light of the novel anorectic effects of OEA, this trial was designed to investigate whether dietary PE may also enhance satiety. To determine whether PE-containing PLs affect appetite regulation, a high-fat test-breakfast containing zero, medium or high doses of PE were given to a group of healthy male participants. Hunger ratings were measured through the morning and EI covertly measured at a buffet style meal presented for lunch.

## Results

### Subjects

Eighteen lean, healthy men completed this 4 treatment trial. Baseline characteristics are provided in Table 1. There was no significant difference in body weight at baseline between the 4 treatments nor was there a change in body weight, BMI or waist circumference throughout the trial (data not shown). There was no significant difference in mean reported EI (high-PE = 9664, kJ 717 sem; medium-PE = 8782 kJ, 511 sem; no-PE control lipid = 9685 kJ, 485 sem; water control = 8877 kJ, 784 sem; P > 0.05) or mean physical activity level (high-PE = 3.8, 0.7 sem; medium-PE = 3.2, 0.5 sem; no-PE control lipid = 4.0, 0.8 sem; water control = 5.0, 1.2 sem; hours spent on mild-moderate activity, P > 0.05) on the day prior to each treatment visit.

**Table 1:** Baseline characteristics of the 18 males who completed the intervention.

	Mean	sd
n	18	
Age (y)	25	7.5
Body weight (kg)	70.7	8.3
BMI (kg/m^2^)	22.1	2.2
Waist circumference (cm)	77.0	5.2
Total cholesterol (mmol/L)	4.0	0.8
LDL-C (mmol/L)	2.1	0.7
HDL-C (mmol/L)	1.5	0.2
Triglycerides (mmol/L)	0.8	0.4
TC:HDL-C ratio	2.7	0.5
Glucose (mmol/L)	4.4	0.3
SBP (mm Hg)	114	10
DBP (mm Hg)	68	6

All measurements made at screen visit.

BMI, body mass index; LDL-cholesterol, low density lipoprotein cholesterol; HDL-cholesterol, high density lipoprotein cholesterol; TC:HDL-C ratio, Total cholesterol: high density lipoprotein-cholesterol ratio; SBP, systolic blood pressure; DBP, diastolic blood pressure

### Visual analogue scales (VAS)

#### Palatability of the test-breakfast

**Figure 1:**
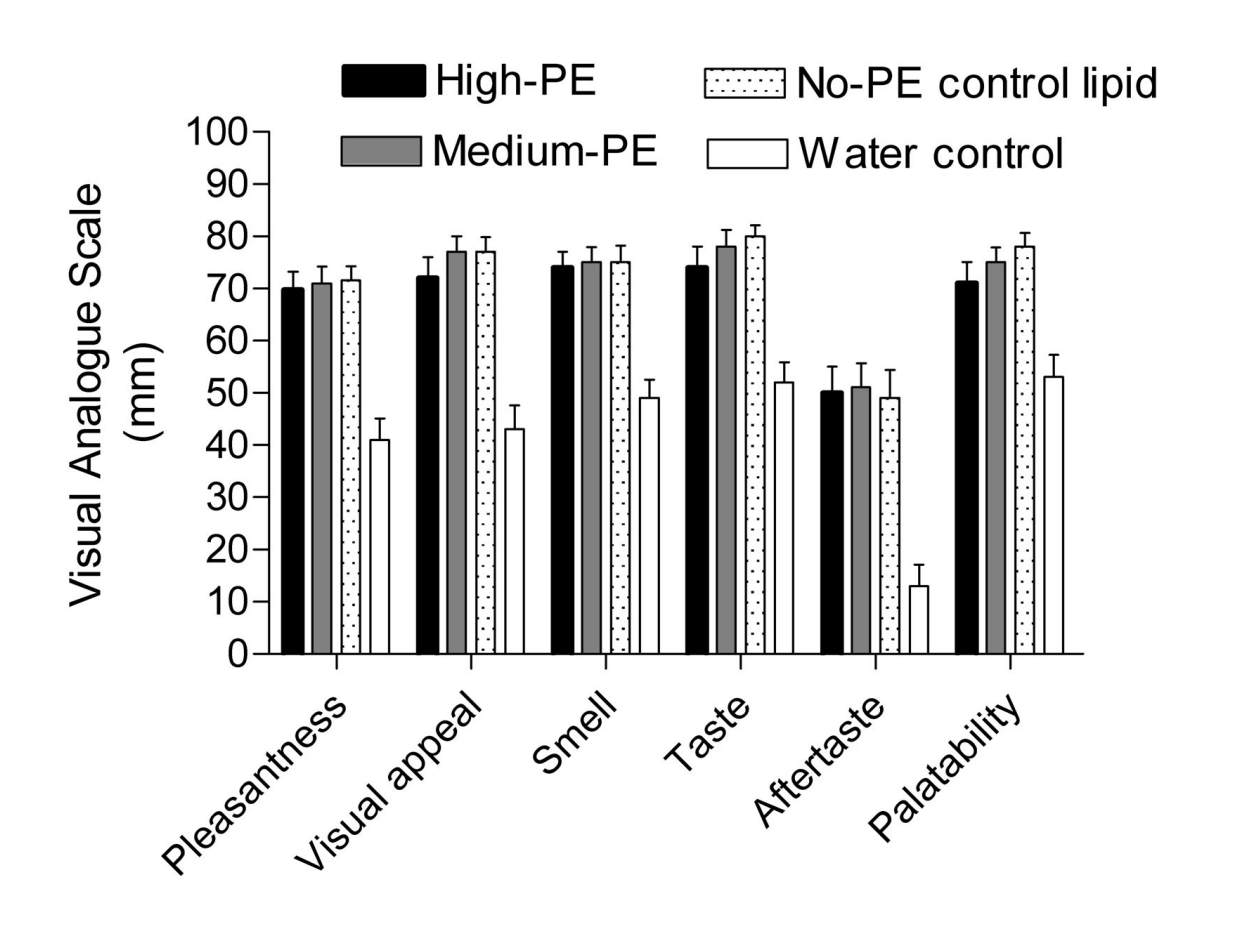
Mean (sem) visual analogue scale (VAS) showing scores for pleasantness, visual appeal, smell, taste, after-taste and palatability of the 4 test-breakfasts.

The lipid containing test-breakfasts (high-PE, medium-PE and no-PE control lipid) were designed to be as similar in appearance and taste as possible, with no significant difference in total energy, fat, carbohydrate (CHO) or protein content, as shown in Table 2. Mean ratings for pleasantness, visual appeal, smell, taste, after-taste and palatability of the 3 lipid containing test-breakfasts and the water control were self-reported through completion of VAS immediately after the breakfast on each occasion, as shown in Figure 1. There was a significant effect of treatment on measures of pleasantness, visual appeal, smell, taste, aftertaste and palatability when all 4 treatments were included in the analysis (P < 0.001), a consequence of inclusion of the water control treatment in the ANOVA. When *post-hoc *analyses were carried out, there was no significant difference between the 3 lipid-containing treatments in pleasantness, visual appeal, smell, taste, after-taste and palatability (P > 0.05) demonstrating that perceived differences in the test-breakfast were not responsible for any differences in EI that may later occur at the *ad libitum *lunch. There was also no significant difference when the 2 PE treatments were compared with the no-PE control lipid (P > 0.05).

**Table 2:** Composition of the four test-breakfasts

	High-PE lipid	Medium-PE lipid	No-PE control lipid	Water control
Energy (MJ)	2.48	2.48	2.46	0
Fat (g)	35.6	35.6	35.3	0
Fat (% en)	53.1	53.2	53.0	0
CHO (g)	41.9	41.9	41.6	0
CHO (% en)	27.0	27.1	27.1	0
Protein (g)	25.8	25.8	25.8	0
Protein(% en)	17.7	17.7	17.9	0
PL (g)	2.42	1.96	0.10	0
PE (g)	0.68	0.57	0.04	0

PE, phosphatidylethanolamine; PL, phospholipid; CHO, carbohydrate

Energy and macronutrient composition of the four test-breakfasts was calculated using the dietary program FoodWorks™ (Professional Edition, Version 2.10.136, 1998–2000; Xyris Software)

#### Hunger and satiety

**Figure 2:**
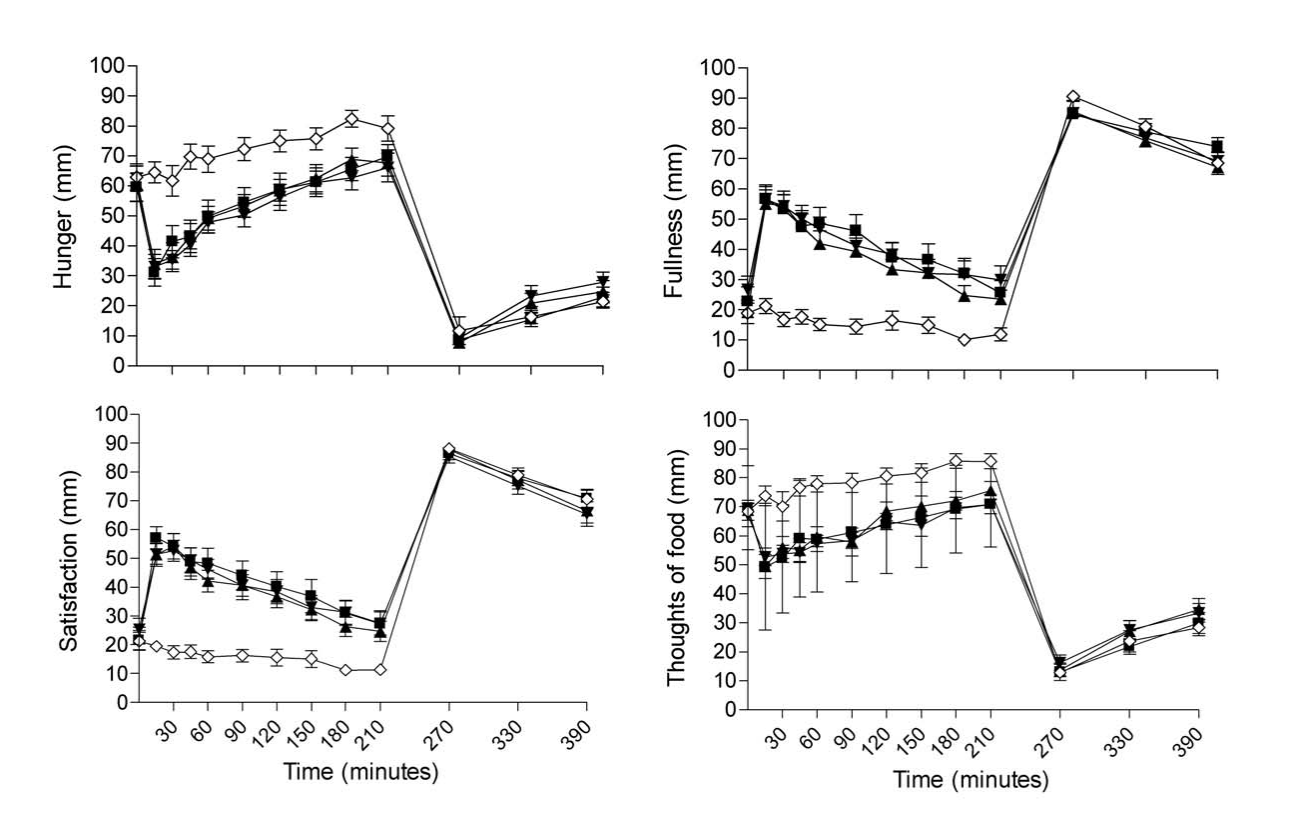
**Mean (sem) visual analogue scale (VAS) showing scores for hunger, fullness, satisfaction and thoughts of food throughout the day.** Lunch was given immediately after VAS assessment at 210 minutes. High-PE (■), Medium-PE (▲), No-PE control lipid (▼), Water control (◇).

The mean VAS ratings for hunger, fullness, satisfaction and thoughts of food measured throughout each study day on the 4 treatment arms are shown in Figure 2. There was no significant difference in hunger, fullness, satisfaction or thoughts of food between any of the treatments at baseline when subjects were fasted. Immediately following the test breakfast hunger rapidly decreased and fullness increased in all 3 lipid treatments in response to the energy bolus. By 210 minutes (prior to the *ad lib *lunch) both hunger and fullness had gradually returned to close to fasting levels. There was no change in VAS measures for the water control. As expected, when analyzed over the 3.5 h post-test-breakfast period, there was a significant effect of the 4 treatments on hunger, fullness, satisfaction and thoughts of food (treatment, P < 0.001), again a consequence of inclusion of the no-energy water control in the analyses. There was also a significant difference between the treatments over time (treatment*time, interaction, P < 0.001). When *post-hoc *analyses were carried out and the two PE treatments combined and compared with water control, there was a significant difference for all 4 VAS measures between treatments (treatment, P < 0.05), but there was no significant difference in hunger, fullness, satisfaction or thoughts of food when compared with the no-PE control lipid (P > 0.05). When the high-PE was compared directly with the medium-PE treatment, there was also no significant difference in hunger, fullness, satisfaction or thoughts of food as measured by VAS (P > 0.01).

### *Ad libitum *lunch

**Figure 3:**
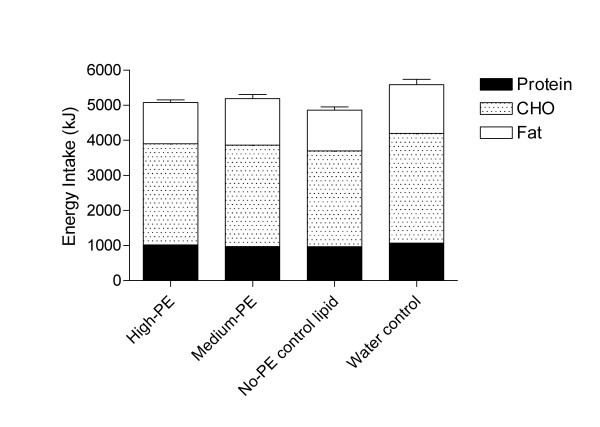
Mean (sem) energy and macronutrient intake at the *ad libitum *lunch.

The mean energy and macronutrient intakes and the total weight of food consumed at the *ad libitum *lunch are presented in Table 3. As expected, there was a significant difference in EI at lunch between the 4 treatments (P < 0.05), driven primarily by the high intake following the 0 kJ water control treatment. Energy intake was 503 kJ, 385 kJ and 719 kJ lower than for the water control when the high-PE, medium-PE or no-PE (neutral lipid control) lipid treatments respectively were given. It was notable that complete or even near complete compensation for the 2.5 MJ breakfast was not achieved on any lipid treatment. Greatest compensation occurred on the no-PE neutral lipid control and was only 29% of the energy provided in the breakfast. *Post-hoc *analyses revealed that significantly greater energy was consumed at lunch when the two PE lipid treatments (5347 kJ, 204 sem) were combined and compared with the no-PE control lipid (5072 kJ, 262 sem, P < 0.05), however there was no significant difference between the high-PE (5288 kJ, 244 sem) and medium-PE (5406 kJ, 334 sem) treatments. When composition of the lunch was examined across the 4 treatments there were significant differences between the intake of all 3 macronutrients (fat, CHO, protein; all, P < 0.05, Figure 3). This was again driven primarily by the 0 kJ water control where macronutrient intake mirrored EI and was high at the lunch meal. When the two PE treatments were compared with the no-PE control lipid, there was no significant difference in the intake of either fat or protein but there was a significantly greater intake of CHO in the two PE arms (P < 0.05) which lead to the observation of higher EI on these treatments. When the high-PE was compared with the medium-PE treatment, there was no difference in intake of CHO or protein but a difference was present in grams of fat consumed (P < 0.05).

**Table 3:** Energy and macronutrient intake at *ad libitum *lunch

	High-PE lipid	Medium-PE lipid	No-PE control lipid	Water control
Energy (kJ)	5288 (244)	5406 (334)	5072 (262)	5792 (233)
Fat (g)	31.2 (2)	35.2 (3)	31.0 (2)	36.3 (2)
Fat (% en)	22 (0.7)	24 (1.2)	22 (1.0)	23 (0.9)
CHO (g)	183.7 (8.4)	184.1 (11.1)	173.8 (9.7)	197.7 (9.5)
CHO (% en)	56 (1)	55 (1)	58 (1)	55 (1)
Protein (g)	61.0 (5)	58.2 (5)	57.8 (5)	65.3 (4)
Protein (% en)	19.5 (1)	18.3 (1)	19.8 (1)	19.3 (1)
Weight of food (g)	1273 (54)	1231 (69)	1245 (61)	1343 (55)

Mean (sem); PE, phosphatidylethanolamine; CHO, carbohydrate; % en, % of energy

## Discussion

Despite its relationship with the known anorectic lipid OEA, there was no evidence from this intervention that the phospholipid PE altered perceptions of hunger and fullness or decreased food intake. This was a short-term assessment of appetite regulation in a group of lean men who arguably are likely to have good physiological regulation of appetite control. There are several issues that should be considered, including the appropriateness of the study design to detect relatively small changes in appetite control. There are a wide range of published methods by which appetite has been assessed and our current trial methodology was based on well known lipid emulsion trials [9-11]. The buffet lunch was given 3.5 h after the HF test-breakfast and the methodology was shown to be sensitive to changes in energy load for both VAS and *ad lib *intake. High, medium and no PE treatments decreased EI by 9, 7 and 12% respectively compared to the water treatment, equivalent to a decrease of between 385–719 kJ at the buffet lunch. Secondly, to assess whether the buffet style lunch may have masked physiological changes in appetite by encouraging over-consumption at the *ad lib *meal it was possible to compare the predicted daily intake of the participants (~10.2 MJ/d, based upon 1.4 × resting expenditure) with mean intake at the lunch meal and conclude that overeating did not occur to any significant degree. Previous trials have described over-consumption when ~7 MJ was eaten at a single meal in participants with predicted energy requirements comparable to that of the subjects in our current trial [19]. Thirdly, the participants were blinded to the 3 lipid treatments and VAS scores confirmed that there were no detectable differences in pleasantness, visual appeal, smell, taste, after-taste or palatability which may have affected hunger scores or food intake.

Dietary fat is believed to be the least satiating of the 3 food macronutrients [1-3,20], and high-fat diets may give rise to over-consumption due to the fact that fat may be less effective than CHO and protein in signalling satiety [2,21,22]. Preliminary evidence exists which indicates that differential satiating characteristics of lipids may be driven by physicochemical properties such as fatty acid saturation [5,7], fatty acid chain length [8] and fat emulsification [9-12]. For example some studies have demonstrated that PUFA may exert relatively stronger control over appetite than MUFA or SFA[5,7], although this is not a universal finding [23,24]. This has been purported to be the consequence of an oxidative gradient whereby PUFA and MUFA may be oxidized more rapidly than SFA [25-27]. There is also evidence that fatty acid chain length may affect satiety, with data from early animal [28] and human [8] studies where EI was decreased on a diet enriched with high medium chain (MCT) versus long chain triglyceride (LCT). In addition Burns and colleagues have demonstrated that lipid/water emulsions may significantly decrease energy and macronutrient intake at a single meal [10] and over 24 hours [9] when compared with isoenergetic non-emulsified lipid. These studies have recently been supported by longer term data showing improved weight maintenance in overweight participants administered a lipid emulsion [29].

Fats and oils are neutral compounds. Replacement of a fatty acid within a triglyceride by a phosphate group results in a PL with 2 non-polar hydrophobic tails and a charged hydrophilic head. Two fatty acids are esterified to glycerol, which is linked through phosphate to a polar head group which in turn may comprise several different compounds; for example choline, serine or ethanolamine [30]. A variety of biochemically important molecules can be obtained by forming a second ester linkage to the phosphate group and of these phosphate diesters, often called phosphatides, the two most biologically important are PE and PC. PLs are found in the diet in items such as eggs, peanuts, liver, sunflower and soybean oil and the average intake of PL is ~5% (w/w) of total fat consumed. PE is known to play a role in the production of endogenous OEA which is an analogue of the endogenous cannabinoid anandimide [17] and which has been shown to inhibit food intake in starved rats [31]. PE is metabolized endogenously into a range of NAEs which are ethanolamides of long chain fatty acids, and which include *N*-arachidonoylethanolamine (anandamide) and its analogue OEA [14].

Rodriguez de Fonseca and colleagues demonstrated that food deprivation markedly decreased OEA biosynthesis in the small intestine[17], and that intraperitoneal administration of OEA caused a decrease in food intake where it may act locally on sensory fibres in the intestine. Fu and colleagues then demonstrated that OEA binds with high affinity to PPAR-α, a nuclear receptor that regulates several aspects of lipid metabolism [32], and found that administration of OEA induces satiety and decreases body weight gain in wild-type mice, but not in mice deficient in PPAR-α[15]. It was hypothesized that OEA would not be orally active because of putative excessive catabolism in the gastrointestinal tract, where a high level of the enzyme fatty acid amide hydrolase is found [33,34]. However, it has since been shown that oral OEA inhibited food intake dose dependently at 90 minutes after food presentation to starved rats [18]. Food intake was decreased by 15.5% by administration of 10 mg/kg OEA. Certainly OEA has proven itself to be an interesting candidate as a satiating factor and other PLs such as PE may be active in a similar way.

The dose of PE used in our study varied increased across treatments. The neutral lipid control contained little or no PL or PE, medium-PE treatment contained 1.96 g PL and 565.3 mg PE (equivalent to 8.08 mg/kg in a 70 kg individual) and high-PE treatment contained 2.42 g PL and 682.4 mg PE (equivalent to 9.75 mg/kg in a 70 kg individual). These doses were comparable with that of Rodriguez de Fonseca and colleagues who administered 5–20 mg/kg intraperitoneally [17] and Nielsen and colleagues who administered 10 mg/kg oral OEA [18]. Interestingly, sensory tests carried out using human participants in our laboratory prior to our current study showed that the relatively unpleasant taste of PL would prevent very high dose lipid administration. In our current study 2.4 g was added to a large 2.5 MJ breakfast in order to mask the distinctive flavour.

## Conclusion

In conclusion, there was no evidence from this single dose trial that PE-containing phospholipids can modulate post-ingestive satiety and energy intake, even at relatively high doses. Whether PE may play a role when administered longer-term is not known.

## Methods

### Subjects

Eighteen healthy, non-smoking male adults were recruited via advertisement. Participants had no self-reported history of significant disease including cardiovascular disease, diabetes, or any other metabolic, endocrine or gastrointestinal disease. All were normotensive and had normal clinical biochemistry as assessed by lipid profile, full blood count and fasting blood glucose. None of the participants were taking medications which may have had an effect on appetite or weight regulation throughout the trial. Written consent was obtained from each participant and ethical approval obtained from the Northern Regional Ethics Auckland, New Zealand.

### Study design

This was a covert manipulation of dietary lipids which varied in their content of the phospholipid, PE. All participants in this randomised, cross-over were required to attend the Human Nutrition Unit on 4 occasions, each for a single day. Between treatment visits, participants returned home for a minimum of 5 days washout with the exception of 1 subject who completed his last visit with only a 1 day washout between treatments. During the washout period participants were requested not to make significant changes to their usual diet and exercise patterns wherever possible. Participants were also asked to abstain from alcohol and strenuous physical activity for 24 hours prior to the study-day, and to fast from 2000 h the previous evening. They completed a 24 h diet recall and 24 h physical activity questionnaire for the day prior to each treatment as a prompt to these requests. Participants were requested to arrive at the Unit using motorized transport and avoid morning exercise. Upon arrival, body weight and waist circumference were measured and any adverse events or medications were recorded. They were then given 200 ml water and completed the first series of visual analogue scales (VAS) which rated their subjective feelings of hunger, fullness, satisfaction and thoughts of food [35]whilst fasted. The test-breakfast was served at 0830 h which was consumed in full within 15 minutes. Participants remained within the Unit throughout each treatment day and were allowed to read, write or undertake other sedentary activities but were not allowed to sleep.

### Test-breakfast

On each study-day, participants received one of 4 test-breakfasts in randomised order: (i) high-PE phospholipid, (ii) medium-PE phospholipid, (iii) no-PE control neutral lipid, (iv) water control. Each of the 3 lipid treatments were matched for energy (2.5 MJ) and fat content (53 en%). The 0 kJ water control was used to confirm that the test system was sensitive to changes in energy. The test-breakfasts comprised 2 savoury muffins plus 300 ml water, or a 300 ml water control. The breakfasts were designed using the dietary program FoodWorks™ (Professional Edition, Version 2.10.136, 1998–2000; Xyris Software) and the macronutrient composition, PL and PE content of the 4 breakfasts is shown in Table 2.

### Dose of phospholipid and PE in the test-breakfasts

The high-PE treatment contained 2.42 g PL, of which 682.4 mg was PE which was equivalent to 9.75 mg/kg in a 70 kg individual. The medium-PE treatment contained 1.96 g PL, of which 565.3 mg was PE which was equivalent to 8.08 mg/kg in a 70 kg individual. Equivalent dose levels in animal trials have been shown to decrease spontaneous EI in rats [17,18]

### Visual analogue scales

VAS were used according to the standard methodology of Flint and colleagues [35]. The following questions were used: "How hungry do you feel?/How full do you feel?/How satisfied do you feel?/How much do you think you can eat now?" and were anchored on the left by "I am not hungry/I am not full at all/I am completely empty/nothing at all" and "I am as hungry as I have ever been/I am totally full/I cannot eat another bite/a large amount" on the right. A set of scales rating how thirsty, energetic and relaxed the participants felt were included as a distraction from the main outcome. Participants were asked to mark their responses by placing a vertical line across the 100-mm scale according to their subjective feelings. Identical VAS questions presented on a separate page each time were completed at baseline, 15, 30, 45, 60, 90, 120, 150, 180, 210, 270, 330, and 390 minutes after the subject began eating the test-breakfast. Immediately after the test-breakfast, participants also rated the pleasantness, visual appeal, smell, taste, aftertaste and overall palatability of the test-breakfast on separate 100-mm VAS.

### *Ad libitum *lunch

The *ad libitum *lunch consisted of a restricted buffet-style meal with predominantly cold and one hot meal choice, along with a selection of beverages as shown in Table 4. Prior to the study, it had been established with each subject that the items provided in the lunch were acceptable as meal choices. In an attempt to avoid over-consumption the variation of the items presented was limited. Each meal item was served in excess and was covertly weighed before and after the meal for calculation of energy and macronutrient intake. Participants were served lunch at 1200 h, which was 3.5 hours after the test-breakfast, in quiet, individual dining rooms and told that they could eat as much or as little as they chose from the free selection over a 45 minute period. The study design, including the long interval of 3.5 hours between the treatment and lunch and the free choice buffet provided at the lunch, was based on the trials of Livingstone and colleagues where anorectic effects have been observed following administration of lipid emulsions [9-11].

**Table 4:** Energy and macronutrient composition of foods and beverages served at the *ad libitum *lunch

Food and beverage item	Portion size (g)	Energy (kJ)	Protein (g)	Fat (g)	CHO (g)
Fried rice	924	4623	38.9	29.9	167.3
Bread, rye	168	1780	13.3	3.2	82.0
Bread, white	168	1814	13.6	3.2	83.8
Chicken breast, roasted	190	1170	48.1	9.8	0
Ham, smoked	190	868	30.4	3.8	13.3
Capsicum green, raw	68	45	0.6	0.3	1.5
Capsicum red, raw	68	99	1.2	0.1	4.6
Tomatoes, raw	127	86	1.1	0.3	3.4
Spiced apple & fruit loaf	400	5520	20.0	45.2	212.4
Apricot pieces tinned in fruit juice, drained	560	1125	3.4	0	45.9
Butter, spreadable	250	6458	2.5	175.0	3.5
Mayonnaise	250	3675	2.0	76.8	44.5
Soy sauce	300	297	3.0	0	15.0
Milk, full fat	1000	2550	31.0	33.0	47.0
Cola drink	1635	2943	0	0	178.2
Orange juice	1000	1820	6.0	2.0	100
Water, bottled	1500	0	0	0	0

Decaffeinated coffee and teas were also available

### Statistical analysis

Energy, macronutrient intake and the weight of the food consumed at the *ad libitum *lunch were analysed using the dietary program FoodWorks™ (Professional Edition, Version 2.10.136, 1998–2000; Xyris Software). Single factor ANOVA (Microsoft Office Excel, 2003) was used to determine any differences in EI or physical activity level on the day prior to each treatment visit. Single factor ANOVA (Microsoft Office Excel, 2003) was also used to identify any differences in body weight at baseline between the 4 treatments or any changes in body weight, BMI or waist circumference throughout the trial. VAS data were analysed using repeated measures Linear Mixed Model ANOVA (SAS: PROC MIXED, SAS version 8.0, SAS Institute Inc, Cary, NC, 2001). The subject, the dietary treatment, the intervention period, and the study day were included in the procedure, as was the treatment/time interaction which addressed whether the trajectory over time during the intervention period differed between treatments (diet*time). *Ad libitum *lunch data was analysed using univariate ANOVA (SAS: PROC MIXED, SAS version 8.0, SAS Institute Inc, Cary, NC, 2001). Statistical significance was based on 95% limits (P < 0.05).

## List of abbreviations

CHO: carbohydrate; EI: energy intake; HF: high fat; LCT: long chain triglyceride; MCT: medium chain triglyceride; MUFA: monounsaturated fatty acids; NAE: *N*-acylethanolamine; OEA: oleoylethanolamide; PE: phosphatidylethanolamine; PC: phosphatidylcholine; PL: phospholipid; PPAR-α: polyunsaturated fatty acids; PUFA: peroxisome-proliferator-activated receptor-α; SFA: saturated fatty acids; VAS: visual analogue scale

## Competing interests

The authors declare that they have no competing interests.

## Authors' contributions

FEL protocol supervision, patient recruitment, registration, data collection, data entry, co-senior author. CMS data collection, data entry, manuscript preparation. ATM physician, clinical oversight of the participants, manuscript preparation. AKM development and preparation of the PE product. BHM biostatistician. SDP fundraiser, protocol design, study oversight, co-senior author.

## References

[B1] Blundell JE, King NA, DJ C (1996). Overconsumption as a cause of weight gain: behavioural-physiological interactions in the control of food intake (appetite). Ciba Foundation Symposium.

[B2] Blundell JE, MacDiarmid JI (1997). Fat as a risk factor for overconsumption: satiation, satiety, and patterns of eating. J Am Diet Assoc.

[B3] Holt SH, Delargy HJ, Lawton CL, Blundell JE (1999). The effects of high-carbohydrate vs high-fat breakfasts on feelings of fullness and alertness, and subsequent food intake. Int J Food Sci Nutr.

[B4] Rolls BJ, Hammer VA (1995). Fat, carbohydrate, and the regulation of energy intake. Am J Clin Nutr.

[B5] French SJ, Conlon CA, Mutuma ST, Arnold M, Read NW, Meijer G, Francis J (2000). The effects of intestinal infusion of long-chain fatty acids on food intake in humans. Gastroenterology.

[B6] Kamphuis MM, Westerterp-Plantenga MS, Saris WH (2001). Fat-specific satiety in humans for fat high in linoleic acid vs fat high in oleic acid. Eur J Clin Nutr.

[B7] Lawton CL, Delargy HJ, Brockman J, Smith FC, Blundell JE (2000). The degree of saturation of fatty acids influences post-ingestive satiety. Br J Nutr.

[B8] Stubbs RJ, Harbron CG (1996). Covert manipulation of the ratio of medium to long-chain triglycerides in isoenergetically dense diets: effect on food intake in ad libitum feeding men. Int J Obes Relat Metab Disord.

[B9] Burns AA, Livingstone MB, Welch RW, Dunne A, Reid CA, Rowland IR (2001). The effects of yoghurt containing a novel fat emulsion on energy and macronutrient intakes in non-overweight, overweight and obese subjects. Int J Obes Relat Metab Disord.

[B10] Burns AA, Livingstone MB, Welch RW, Dunne A, Robson PJ, Lindmark L, Reid CA, Mullaney U, Rowland IR (2000). Short-term effects of yoghurt containing a novel fat emulsion on energy and macronutrient intakes in non-obese subjects. Int J Obes Relat Metab Disord.

[B11] Burns AA, Livingstone MB, Welch RW, Dunne A, Rowland IR (2002). Dose-response effects of a novel fat emulsion (Olibra) on energy and macronutrient intakes up to 36 h post-consumption. Eur J Clin Nutr.

[B12] Welch IM, Sepple CP, Read NW (1988). Comparisons of the effects on satiety and eating behaviour of infusion of lipid into the different regions of the small intestine. Gut.

[B13] Cunnane S, Griffin B, Gibney MVH, Kok F (2002). Nutrition and metabolism of lipids. Introduction to Human Nutrition.

[B14] Sun Y-X, Tsuboi K, Okamoto Y, Tonai T, Murakami M, Kudo I, Ueda N (2004). Biosynthesis of anandamide and N-palmitoylethanolamine by sequential actions of phospholipase A2 and lysophospholipase D. Biochem J.

[B15] Fu J, Gaetani S, Oveisi F, Lo Verme J, Serrano A, Rodriguez De Fonseca F, Rosengarth A, Luecke H, Di Giacomo B, Tarzia G, Piomelli D (2003). Oleylethanolamide regulates feeding and body weight through activation of the nuclear receptor PPAR-alpha. Nature.

[B16] LoVerme J, Gaetani S, Fu J, Oveisi F, Burton K, Piomelli D (2005). Regulation of food intake by oleoylethanolamide. Cell Mol Life Sci.

[B17] Rodriguez de Fonseca F, Navarro M, Gomez R, Escuredo L, Nava F, Fu J, Murillo-Rodríguez E, Giuffrida A, Lo Verme J, Gaetani S, Kathuria S, Gall C, Piomelli D (2001). An anorexic lipid mediator regulated by feeding. Nature.

[B18] Nielsen MJ, Petersen G, Astrup A, Hansen HS (2004). Food intake is inhibited by oral oleoylethanolamide. J Lipid Res.

[B19] Arvaniti K, Richard D, Tremblay A (2000). Reproducibility of energy and macronutrient intake and related substrate oxidation rates in a buffet-type meal. Br J Nutr.

[B20] Rolls BJ, Castellanos VH, Halford JC, Kilara A, Panyam D, Pelkman CL, Smith GP, Thorwart ML (1998). Volume of food consumed affects satiety in men. Am J Clin Nutr.

[B21] Blundell JE, Lawton CL, Hill AJ (1993). Mechanisms of appetite control and their abnormalities in obese patients. Horm Res.

[B22] Green SM, Burley VJ, Blundell JE (1994). Effect of fat- and sucrose-containing foods on the size of eating episodes and energy intake in lean males: potential for causing overconsumption. Eur J Clin Nutr.

[B23] Flint A, Helt B, Raben A, Toubro S, Astrup A (2003). Effects of different dietary fat types on postprandial appetite and energy expenditure. Obes Res.

[B24] MacIntosh CG, Holt SH, Brand-Miller JC (2003). The degree of fat saturation does not alter glycemic, insulinemic or satiety responses to a starchy staple in healthy men. J Nutr.

[B25] Kien CL, Bunn JY, Ugrasbul F (2005). Increasing dietary palmitic acid decreases fat oxidation and daily energy expenditure. Am J Clin Nutr.

[B26] Piers LS, Walker KZ, Stoney RM, Soares MJ, O'Dea K (2002). The influence of the type of dietary fat on postprandial fat oxidation rates: monounsaturated (olive oil) vs saturated fat (cream). Int J Obes Relat Metab Disord.

[B27] Soares MJ, Cummings SJ, Mamo JC, Kenrick M, Piers LS (2004). The acute effects of olive oil v. cream on postprandial thermogenesis and substrate oxidation in postmenopausal women. Br J Nutr.

[B28] Friedman MI, Edens NK, Ramirez I (1983). Differential effects of medium- and long-chain triglycerides on food intake of normal and diabetic rats. Physiol Behav.

[B29] Diepvens K, Soenen S, Steijns J, Arnold M, Westerterp-Plantenga M (2007). Long-term effects of consumption of a novel fat emulsion in relation to body-weight management. Int J Obes Relat Metab Disord.

[B30] Zubay G (1993). Biochemistry.

[B31] Hansen HS, Moesgaard B, Hansen HH, Petersen G (2000). N-Acylethanolamines and precursor phospholipids – relation to cell injury. Chem Phys Lipids.

[B32] Schoonjans K, Staels B, Auwerx J (1996). The peroxisome proliferator activated receptors (PPARs) and their effects on lipid metabolism and adipocyte differentiation. Biochim Biophys Acta.

[B33] Cravatt BF, Lichtman AH (2002). The enzymatic inactivation of the fatty acid amide class of signaling lipids. Chem Phys Lipids.

[B34] Katayama K, Ueda N, Kurahashi Y, Suzuki H, Yamamoto S, Kato I (1997). Distribution of anandamide amidohydrolase in rat tissues with special reference to small intestine. Biochim Biophys Acta.

[B35] Flint A, Raben A, Blundell JE, Astrup A (2000). Reproducibility, power and validity of visual analogue scales in assessment of appetite sensations in single test meal studies. Int J Obes Relat Metab Disord.

